# Transition of *bla*_OXA-58-like_ to *bla*_OXA-23-like_ in *Acinetobacter baumannii* Clinical Isolates in Southern China: An 8-Year Study

**DOI:** 10.1371/journal.pone.0137174

**Published:** 2015-09-04

**Authors:** Weiyuan Wu, Yi He, Jian Lu, Yuemei Lu, Jinsong Wu, Yingxia Liu

**Affiliations:** 1 Department of Laboratory Medicine, Shenzhen People’s Hospital, Second Clinical Medical College of Jinan University, Key Laboratory of Pathogenic Microorganism and Bacterial Resistance Surveillance in Shenzhen, Shenzhen, Guangdong, China; 2 Department of Infectious Disease, Third People’s Hospital of Shenzhen, Shenzhen, Guangdong, China; The University of Sydney, AUSTRALIA

## Abstract

**Background:**

The prevalence of carbapenem-resistant *Acinetobacter baumannii* in hospitals has been increasing worldwide. This study aims to investigate the carbapenemase genes and the clonal relatedness among *A*. *baumannii* clinical isolates in a Chinese hospital.

**Methods:**

Carbapenemase genes and the upstream locations of insertion sequences were detected by polymerase chain reaction (PCR), and the clonal relatedness of isolates was determined by pulsed-field gel electrophoresis (PFGE) and multilocus sequence typing.

**Results:**

A total of 231 nonduplicate carbapenemase gene-harboring *A*. *baumannii* clinical isolates recovered from Shenzhen People’s Hospital, were investigated between 2002 and 2009. *bla*_OXA-23-like_, *bla*_OXA-58-like_, *bla*_OXA-40-like_, and IS*Aba1*-*bla*_OXA-51-like_ were identified in 119, 107, 1, and 4 isolates, respectively. IS*1008*-ΔIS*Aba3*, IS*Aba3*, and IS*Aba1* were detected upstream of the *bla*_OXA-58-like_ gene in 69, 35, and 3 isolates, respectively. All *bla*_OXA-23-like_ genes but one had an upstream insertion of IS*Aba1*. *bla*_OXA-58-like_ was the most common carbapenemase gene in *A*.*baumannii* before 2008, thereafter *bla*_OXA-23-like_ became rapidly prevalent and replaced *bla*_OXA-58-like_ in 2009. The majority of *bla*_OXA-58-like_-carrying isolates showed lower level of resistance to imipenem and meropenem (minimum inhibitory concentrations (MICs), 1 μg/ml to 16 μg/ml), compared with the majority of *bla*_OXA-23-like_-carrying isolates (MICs, 16 μg/ml to 64 μg/ml for both imipenem and meropenem). All 231 *bla*_OXA_ carbapenemase gene-harboring isolates belonged to 14 PFGE types (A–N), and three dominant clones A, J, and H accounted for 43.3%, 42.0%, and 8.2% of the tested isolates, respectively. Clone A (sequence type ST92/ST208) with *bla*_OXA-58-like_ was the most prevalent before 2008. Clone H (ST229) with *bla*_OXA-23-like_ became striking between 2007 and 2008. Clone J (ST381) with *bla*_OXA-23-like_ rapidly spread and replaced clones A and H in 2009.

**Conclusion:**

This study is the first to reveal that the distinct *bla*_OXA-23-like_-carrying *A*. *baumannii* ST381 displaced the previously prevalent *bla*_OXA-58-like_-carrying *A*. *baumannii* ST92/ST208, resulting in the rapidly increasing resistance to carbapenems in *A*. *baumannii* in Shenzhen People’s Hospital in 2009.

## Introduction

Over the past decade, increasing resistance to carbapenems in *Acinetobacter baumannii* has been observed worldwide [1]. This increasing resistance is mainly mediated by production of class D (carbapenem-hydrolyzing oxacillinases [CHDLs]) β-lactamases (OXAs) with carbapenemase activity. Currently, six OXAs with carbapenemase activity gene clusters have been described in *A*. *baumannii*, including *bla*_OXA-23-like_, *bla*_OXA-40-like_, *bla*_OXA-51-like_, *bla*_OXA-58-like_, *bla*_OXA-143-like_, and *bla*_OXA-235-like_ genes [2, 3]. Although the hydrolytic efficiencies of these OXA carbapenemases for carbapenems are relatively low [4], various insertion sequences (ISs) upstream of the *bla*_OXA_ carbapenemase genes, including IS*Aba1*, IS*Aba2*, IS*Aba3*, IS*18*, IS*125*, IS*1008*, and IS*Aba4*, provide promoters for the expression of *bla*_OXA_ carbapenemase genes, except for *bla*_OXA-40-like_ and *bla*_OXA-143-like_ genes, and mediate resistance to carbapenems [5–9].

Clonal spread of carbapenem-resistant *A*. *baumannii* has been reported worldwide. Three epidemic lineages of *A*. *baumannii*, commonly referred to as the pan-European clonal lineages (EU I, EU II, and EU III), account for the majority of *A*. *baumannii* infections. Strains that belong to EU II (global clone 2) are widespread throughout the world, including China; many epidemiological studies reported the widespread of OXA-58 producers and OXA-23 producers within this lineage [10–12].

In this study, the transition of *bla*_OXA-58-like_ to *bla*_OXA-23-like_ in *A*. *baumannii* clinical isolates from a Chinese hospital between 2002 and 2009 was confirmed. The clonal relatedness of isolates was also investigated by pulsed-field gel electrophoresis (PFGE) and multilocus sequence typing (MLST).

## Material and Methods

### Bacterial isolates and antimicrobial susceptibility testing

All nonduplicate clinical isolates of *A*. *baumannii* were recovered from various wards and clinical samples in Shenzhen People’s Hospital (Shenzhen, Guangdong Province, China), a tertiary-care hospital with 1200 beds, over an 8-year period from 2002 to 2009. The isolates were initially identified using the Vitek 2 system (bioMerieux) and assigned to the *Acinetobacter calcoaceticus*–*A*. *baumannii* complex. Identification of *A*. *baumannii* was confirmed by the presence of *bla*_OXA-51-like_ intrinsic to this species by using PCR [13–15]. Agar dilution was performed to detect susceptibilities to imipenem and meropenem for all *A*. *baumannii* isolates [16]. Isolates with imipenem and/or meropenem minimum inhibitory concentrations (MICs) ≥ 0.25 μg/ml were further investigated for the carbapenemase genes. MICs of other 13 antimicrobial agents were also determined by agar dilution for carbapenemase gene-carrying *A*. *baumannii* isolates. *Escherichia coli* ATCC 25922 and *Pseudomonas aeruginosa* ATCC 27853 were used as the controls.

### Detection of carbapenemase genes and ISs upstream of CHDL genes

PCR assays for genes coding for known carbapenemases (i.e., *bla*_IMP_, *bla*_VIM_, *bla*_SPM_, *bla*_SIM_, *bla*_GIM_, *bla*_OXA-23-like_, *bla*_OXA-40-like_, *bla*_OXA-58-like_, *bla*_OXA-143-like_, and *bla*_KPC_) were performed as previously described [2, 17–19]. PCR with primers within the ISs (i.e., IS*Aba1*, IS*Aba2*, IS*Aba3*, IS*18*, IS*125*, IS*1008*, and IS*Aba4*) and reverse primers within the CHDL genes [5–9] mapped the upstream locations of ISs.

### PFGE

PFGE determined the clonal relationships of the carbapenemase gene-carrying *A*. *baumannii* isolates. PFGE of *Apa*I (New England)-digested genomic DNA was conducted using the GenePath system (Bio-Rad) as previously described [20, 21]. DNA macrorestriction patterns were interpreted according to the criteria described by Tenover et al [22] and cluster analysis was performed using Fingerprint II software (Bio-Rad). Dendrograms for similarity were constructed using the unweighted-pair group method with arithmetic averages. The Dice correlation coefficient was used to analyze any similarities between banding patterns. In brief, isolates that showed zero to three DNA fragment differences and a similarity of ≥ 85% following dendrogram analysis were considered to represent the same PFGE type.

### MLST

MLST was conducted as previously described [11, 23] for the representative isolates from the prevalent main clones typed by PFGE. In brief, internal fragments of seven housekeeping genes, i.e., *gltA*, *gyrB*, *gdhB*, *recA*, *cpn60*, *gpi*, and *rpoD*, were PCR amplified, purified, and then sequenced with an ABI prism sequencer 3730 (Applied Biosystems). A new primer pair was redesigned (*recA*-F2, 5′-GCAGTTGAAGCCGTATCT-3′ and *recA*-R2, 5′-TTGACCGATACGACGAA-3′) for both amplification and sequencing to obtain the specific PCR products and satisfactory sequencing results. The internal fragments for analysis were still identical to a previous scheme [23]. The sequence of each allele was compared by Basic Local Alignment Search Tool with existing sequences in Pubmlst database and sequence types (STs) were designated according to the allelic profiles (http://pubmlst.org/abaumannii/).

## Results

### Distribution of carbapenemase genes

During the study period, 393 nonduplicate clinical isolates of *A*. *baumannii*, with imipenem and/or meropenem MICs ≥ 0.25 μg/ml, were recovered from 367 colonized or infected inpatients in Shenzhen People’s Hospital. A total of 231 isolates of *bla*_OXA_ carbapenemase gene-harboring *A*. *baumannii* were detected among of them. *bla*_OXA-23-like_, *bla*_OXA-58-like_, *bla*_OXA-40-like_, and IS*Aba1*-*bla*_OXA-51-like_ were identified in 119, 107, 1, and 4 single isolates, respectively. *bla*_OXA-143-like_, *bla*_KPC_ genes, and metallo-β-lactamase genes undetected in any isolates identified in this study. *bla*_OXA-58-like_ had been the most common carbapenemase gene in *A*. *baumannii* prior to 2008; thereafter, *bla*_OXA-23-like_ remarkably increased and became rapidly prevalent in *A*. *baumannii* in 2009 (Table 1). IS*1008*-ΔIS*Aba3*, IS*Aba3*, and IS*Aba1* were found upstream of the *bla*_OXA-58-like_ gene in 69, 35, and 3 isolates, respectively. All *bla*_OXA-23-like_ genes but one had an upstream insertion of IS*Aba1*.

**Table 1:** *A*. *baumannii* (Ab) isolates with imipenem and/or meropenem MICs ≥ 0.25 μg/ml from 2002 to 2009.

Organism	No. of isolates	2002	2003	2004	2005	2006	2007	2008	2009
***bla***_**OXA-58-like**_ **-carrying Ab**	107 (90)^a^	4	32	26	8	13	12	7	5
***bla***_**OXA-23-like**_ **-carrying Ab**	119 (116)				1	3	6	8	101
***bla***_**OXA-40-like**_ **-carrying Ab**	1 (1)						1		
**IS*Aba1*-*bla***_**OXA-51-like**_ **-carrying Ab**	4 (4)							1	3
**Noncarbapenemase gene-carrying Ab**	162 (156)	36	20	11	32	16	16	18	13
**Total**	393 (367)	40	52	37	41	32	35	34	122

^*a*^ Parentheses refer to the number of patients

### Antibiotic resistance profiles

Table 2 shows the MIC distributions for both imipenem and meropenem and *bla*_OXA_ carbapenemase gene-harboring *A*. *baumannii*. The majority of *bla*_OXA-58-like_-carrying isolates showed lower level of resistance to imipenem (MICs, 1 μg/ml to 16 μg/ml) and meropenem (MICs, 1 μg/ml to 8 μg/ml), compared with *bla*_OXA-23-like_-carrying isolates (MICs, 16 μg/ml to 64 μg/ml for both imipenem and meropenem). Notably, 26 (24.3%) and 27 (25.2%) isolates with *bla*_OXA-58-like_ were classified as “susceptible” (MICs, 0.5 μg/ml to 2 μg/ml) and “intermediate” to imipenem, respectively, using the current Clinical Laboratory Standard Institute (CLSI) breakpoint for susceptibility of ≤ 2μg/ml and resistance of ≥ 8 μg/ml. Furthermore, higher susceptible rate of 35.5% (38/107) and intermediate rate of 42.1% (45/107) were observed to meropenem against *bla*_OXA-58-like_-carrying *A*. *baumannii* isolates. Only 54 (50.5%) and 24 (22.4%) of 107 *bla*_OXA-58-like_-carrying isolates were classified as resistant to imipenem and meropenem, respectively. By contrast, 119 *bla*_OXA-23-like_-carrying isolates were classified as resistant to both imipenem and meropenem. One *bla*_OXA-40-like_-carrying isolate and four IS*Aba1*-*bla*_OXA-51-like_-carrying isolates were classified as intermediate or resistant to imipenem and meropenem. Majority of *A*. *baumannii* isolates without carbapenemase gene were classified as susceptible to imipenem and meropenem, except for the two (1.2%) and four (2.5%) isolates classified as intermediate to imipenem and meropenem, respectively.

**Table 2:** MIC distributions of imipenem and meropenem against *A*. *baumannii* (Ab) isolates with or without carbapenemase gene.

Organism (no. of isolates tested)	No. of isolates with MIC (μg/ml)										
	0.125	0.25	0.5	1	2	4	8	16	32	64	128
***bla***_**OXA-58-like**_ **-carrying Ab (107)**											
** Imipenem**			2	11	13	27	38	15	1		
** Meropenem**			2	18	18	45	21	2	1		
***bla***_**OXA-23-like**_ **-carrying Ab (119)**											
** Imipenem**								31	36	52	
** Meropenem**							8	24	55	29	3
***bla***_**OXA-40-like**_ **-carrying Ab (1)**											
** Imipenem**							1				
** Meropenem**								1			
**IS*Aba1*-*bla***_**OXA-51-like**_ **-carrying Ab (4)**											
** Imipenem**						1	2	1			
** Meropenem**							2	1	1		
**Noncarbapenemase gene-carrying Ab (162)** ^a^											
** Imipenem**	3	31	18	85	23	2					
** Meropenem**		14	26	72	46	4					

^*a*^ *A*. *baumannii* isolates with imipenem and/or meropenem MICs ≥ 0.25 μg/ml

*bla*_OXA-58-like_-carrying isolates showed moderate susceptibility to a few noncarbapenems (Table 3). More than half of *bla*_OXA-58-like_-carrying isolates were still susceptible or intermediate to cefoperazone-sulbactam, ampicillin-sulbactam, and cefepime, compared with less than 5% of *bla*_OXA-23-like_-carrying isolates. Both *bla*_OXA-58-like_-carrying isolates and *bla*_OXA-23-like_-carrying isolates were highly resistant to piperacillin-tazobactam, ceftazidime, ceftriazone, amikacin, ciprofloxacin, levofloxacin, and trimethoprim-sulfamethoxazole (resistance rates, 75.7% to 100%). However, these isolates all exhibited low resistance to polymixin B, minocycline, and tigecycline (resistance rate of less than 15%). The MIC distributions of imipenem and meropenem for IS*1008*-ΔIS*Aba3*-*bla*_OXA-58-like_-carrying *A*. *baumannii* were similar to those for IS*Aba3*-*bla*_OXA-58-like_-carrying *A*. *baumannii* (Table 4).

**Table 3:** Susceptibilities of 15 antimicrobial agents against *bla*_OXA-58-like_-carrying and *bla*_OXA-23-like_-carrying *A*. *baumannii* (Ab).

Antimicrobial agents	*bla*_OXA-58-like_-carrying Ab (n = 107)						*bla*_OXA-23-like_-carrying Ab (n = 119)					
	R%	I%	S%	MIC_50_ (μg/ml)	MIC_90_ (μg/ml)	MIC Range (μg/ml)	R%	I%	S%	MIC_50_ (μg/ml)	MIC_90_ (μg/ml)	MIC Range (μg/ml)
**Imipenem**	50.5	25.2	24.3	8	16	0.5–32	100	0	0	32	64	16–64
**Meropenem**	22.4	42.1	35.5	4	8	0.5–32	100	0	0	32	64	8–128
**Cefoperazone-sulbactam** ^a^	19.6	14	66.4	16	64	4–128	95.8	2.5	1.7	128	128	16–> 256
**Ampicillin-sulbactam**	36.4	58.9	4.7	16	64	4–128	100	0	0	128	128	32–> 256
**Cefepime**	49.5	46.7	3.7	16	64	2–> 256	99.2	0.8	0	64	128	16–> 256
**Piperacillin-tazobactam**	97.2	1.9	0.9	256	> 256	1–> 256	100	0	0	> 256	> 256	128–> 256
**Ceftazidime**	99.1	0	0.9	> 256	> 256	4–> 256	99.2	0.8	0	> 256	> 256	16–> 256
**Ceftriazone**	94.4	5.6	0	> 256	> 256	16–> 256	98.3	1.7	0	> 256	> 256	16–> 256
**Amikacin**	75.7	15.9	8.4	128	256	1–> 256	82.4	12.6	5	> 256	> 256	1–> 256
**Ciprofloxacin**	97.2	0	2.8	> 32	> 32	0.25–> 32	100	0	0	> 32	> 32	16–> 32
**Levofloxacin**	77.6	18.7	3.7	16	32	0.125–> 32	89.1	10.9	0	16	16	4–32
**Trimethoprim-sulfamethoxazole**	96.3	0	3.7	> 16	> 16	0.125–> 16	99.2	0	0.8	> 16	> 16	0.5–> 16
**Polymixin B**	0	0	100	1	1	0.5–2	0	0	100	1	1	0.5–1
**Minocycline**	12.1	61.7	26.2	8	16	0.125–16	0.8	1.7	97.5	4	4	0.5–16
**Tigecycline** ^b^	10.3	72.9	16.8	4	8	2–16	4.2	79	16.8	4	4	2–8

^*a*^ CLSI (2007) breakpoint for cefoperazone was used for cefoperazone-sulbactam in this study.

^*b*^ U.S. FDA criteria for tigecycline were used in this study (susceptibility is defined as ≤ 2μg/ml; resistance as ≥ 8 μg/ml).

R, resistant; I, intermediate; S, susceptible

**Table 4:** MIC distributions of imipenem and meropenem against *A*. *baumannii* (Ab) isolates with various ISs upstream of the *bla*_OXA-58-like._

Ab with IS upstream of the *bla*_OXA-58-like_	No. of isolates with MIC (μg/ml)						
(no. of isolates tested)	0.5	1	2	4	8	16	32
**IS*1008*-ΔIS*Aba3*-*bla***_**OXA-58-like**_ **(69)**							
** imipenem**		2	5	20	34	8	
** meropenem**		5	11	39	12	1	1
**IS*Aba3*-*bla***_**OXA-58-like**_ **(35)**							
** imipenem**	2	9	8	7	3	5	1
** meropenem**	2	13	7	6	6	1	
**IS*Aba1*-*bla***_**OXA-58-like**_ **(3)**							
** imipenem**					1	2	
** meropenem**					3		

### PFGE and MLST

**Fig 1:**
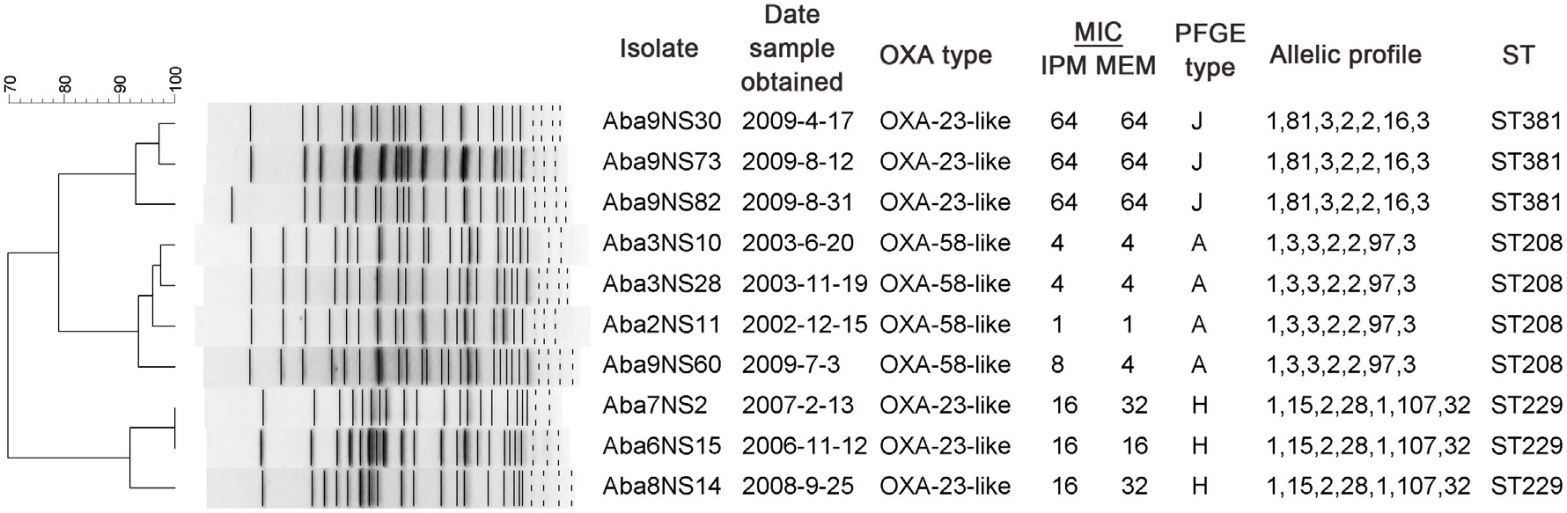
PFGE dendrogram of 10 representative isolates from the three dominant clones. Allelic profile: seven loci in the order *gltA*, *gyrB*, *gdhB*, *recA*, *cpn60*, *gpi*, and *rpoD*; MIC μg/ml; IPM, imipenem; MEM, meropenem.

All *bla*_OXA_ carbapenemase gene-harboring isolates belonged to 14 PFGE types (A–N). Three dominant PFGE-defined clones A, J, and H comprised 100 (43.3%), 97 (42.0%), and 19 (8.2%) isolates, respectively. Clone A with *bla*_OXA-58-like_ had been the most prevalent prior to 2008. Clone H with *bla*_OXA-23-like_ became notable between 2007 and 2008. Clone J with *bla*_OXA-23-like_ rapidly increased and became the dominant clone in place of clones A and H in 2009 (Table 5). Ten representative isolates of the three dominant clones A, J, and H, which were obtained from ten inpatients, belonged to three different sequence types ST92/ST208, ST381, and ST229, respectively. ST229 was different from ST92/ST208 and ST381 by six alleles. Only two allelic (*gyrB* and *gpi*) differences were observed between ST381 and ST92/ST208 (Fig 1), both of which belong to global clone 2.

**Table 5:** PFGE types of carbapenemase gene-carrying *A*. *baumannii* (Ab) from 2002 to 2009.

Year			PFGE type (No.)			
	IS*1008*-ΔIS*Aba3*-*bla*_OXA-58-like_-carrying Ab (69)	IS*Aba3*-*bla*_OXA-58-like_-carrying Ab (35)	IS*Aba1*-*bla*_OXA-58-like_-carrying Ab (3)	*bla*_OXA-23-like_-carrying Ab (119)	IS*Aba1-bla*_OXA-51-like_-carrying Ab (4)	*bla*_OXA-40-like_-carrying Ab (1)
**2002 (n = 4)**		A (3), B (1)				
**2003 (n = 32)**	A (17)	A (13), E (2)				
**2004 (n = 26)**	A (17)	A (8), F (1)				
**2005 (n = 9)**	A (6)	A (1), G (1)		I (1)		
**2006 (n = 16)**	A (13)			H (3)		
**2007 (n = 19)**	A (10)	A (1), C (1)		H (6)		L (1)
**2008 (n = 16)**	A (4)	A (2), D (1)		H (6), I (1), J (1)	I (1)	
**2009 (n = 109)**	A (2)		A (3)	H (4), J (96), K (1)	M (2), N (1)	

ST381 (clone J) showed apparently different resistance profiles compared with ST92/ST208 (clone A) (Table 6). ST381 isolates were uniformly resistant to all β-lactam drugs tested. By contrast, ST92/ST208 isolates showed variable resistance to imipenem, meropenem, cefoperazone-sulbactam, ampicillin-sulbactam, and cefepime.

**Table 6:** Antibiotic resistance profiles of the three main carbapenemase gene-harboring *A*. *baumannii* clones (MIC μg/ml).

		Clone A/ST92/ST208 (n = 100)					Clone J/ST381 (n = 97)					Clone H/ST229 (n = 19)			
Antimicrobial Agents	R%	S%	MIC_50_	MIC_90_	MIC Range	R%	S%	MIC_50_	MIC_90_	MIC Range	R%	S%	MIC_50_	MIC_90_	MIC Range
**IPM**	53	21	8	16	1–32	100	0	64	64	16–64	100	0	16	32	16–64
**MEM**	24	34	4	8	1–32	100	0	32	64	8–128	100	0	32	32	16–64
**CSL** ^a^	20	67	16	64	8–64	100	0	128	128	64–> 256	73.7	10.5	64	64	16–128
**SAM**	37	3	16	64	8–128	100	0	128	256	64–> 256	100	0	64	128	32–128
**FEP**	52	0	32	64	16–128	100	0	64	128	32–> 256	94.7	0	128	128	16–128
**TZP**	100	0	256	> 256	128–> 256	100	0	> 256	> 256	256–> 256	100	0	256	>256	128–> 256
**CAZ**	100	0	> 256	> 256	256–> 256	100	0	> 256	> 256	256–> 256	94.7	0	>256	>256	16–> 256
**CRO**	100	0	> 256	> 256	256–> 256	100	0	> 256	> 256	64–> 256	89.5	0	>256	>256	16–> 256
**AMK**	80	3	128	256	1–> 256	99	1	> 256	> 256	1–> 256	5.3	15.8	32	32	1–64
**CIP**	100	0	64	64	32–64	100	0	64	64	32–64	100	0	32	32	16–64
**LEV**	81	0	16	32	4–64	100	0	16	16	8–32	36.8	0	4	8	4–8
**SXT**	100	0	32	32	4–32	100	0	32	32	32–32	100	0	8	32	4–32
**POL**	0	100	1	1	0.5–2	0	100	1	1	0.5–1	0	100	1	1	0.5–1
**MNO**	12	22	8	16	1–16	0	98	4	4	2–8	0	100	2	2	1–2
**TGC** ^b^	10	14	4	4	2–16	3.1	0	4	4	4–8	0	100	2	2	2–2

IPM, imipenem; MEM, meropenem; CSL, cefoperazone-sulbactam; SAM, ampicillin-sulbactam; FEP, cefepime; TZP, piperacillin-tazobactam; CAZ, ceftazidime; CRO, ceftriaxone; AMK, amikacin; CIP, ciprofloxacin; LEV, levofloxacin; SXT, trimethoprim-sulfamethoxazole; POL, polymixin B; MNO, minocycline; TGC, tigecycline

R, resistant; S, susceptible

^*a*^ CLSI (2007) breakpoint for cefoperazone was used for cefoperazone-sulbactam in this study.

^*b*^ U.S. FDA criteria for tigecycline were used in this study (susceptibility is defined as ≤ 2μg/ml; resistance as ≥ 8 μg/ml).

## Discussion

*bla*_OXA-23-like_ carbapenemase genes are disseminated worldwide [1]. In China, *bla*_OXA-23-like_ is the most common carbapenemase gene in *A*. *baumannii*, with more than 90% of imipenem-nonsusceptible *A*. *baumannii-*harbored *bla*_OXA-23_ [11, 24, 25]. In the present study, 119 (30.1%) and 107 (27.2%) of 393 *A*. *baumannii* isolates with imipenem and/or meropenem MICs ≥ 0.25 μg/ml carried *bla*_OXA-23-like_ and *bla*_OXA-58-like_, respectively. Surprisingly, *bla*_OXA-58-like_ had been the most common carbapenemase gene in *A*. *baumannii* in Shenzhen People’s Hospital until 2008. *bla*_OXA-23-like_ occurred in a sporadic clone I for the first time in the hospital in 2005 and then remarkably increased and became rapidly prevalent in *A*. *baumannii* clone J in 2009. Notably, the similar replacement of *bla*_OXA_ carbapenemase genes in *A*. *baumannii* was reported in Italy during the same period [10, 26]. We also found that the majority of *bla*_OXA-58-like_-carrying isolates showed lower level of resistance to carbapenems compared with *bla*_OXA-23-like_-carrying isolates. Only 54 (50.5%) and 24 (22.4%) of 107 *bla*_OXA-58-like_-carrying isolates were classified as resistant to imipenem and meropenem, respectively, using the current CLSI breakpoint. By contrast, all 119 *bla*_OXA-23-like_-carrying isolates were classified as resistant to both imipenem and meropenem. Less *bla*_OXA-58-like_-carrying isolates would be classified as resistant to imipenem (16/107) and meropenem (3/107) using the previous CLSI breakpoint for susceptibility of ≤ 4 μg/ml and resistance of ≥ 16 μg/ml [27]. Interestingly, *bla*_OXA-58-like_-carrying isolates showed moderate susceptibility to cefoperazone-sulbactam, ampicillin-sulbactam, and cefepime compared with *bla*_OXA-23-like_-carrying isolates, which were highly resistant to these drugs in the present study. Coelho et al. [28] examined 28 isolates of *bla*_OXA-58-like_-carrying *A*. *baumannii* collected worldwide. They found that imipenem and meropenem MICs of 1–4 μg/ml were detected in 17 and 22 isolates, respectively. The carbapenem MICs varied from 32 μg/ml to 1–4 μg/ml for the isolates from different countries. Based on these findings, we speculate that some *bla*_OXA-58-like_-carrying *A*. *baumannii* isolates may spread undetected in previous studies from China because of the relatively low imipenem and/or meropenem MICs for these organisms.

The flanking IS elements IS*Aba1*, IS*Aba2*, IS*Aba3*, IS*Aba825*, IS*18*, and IS*1008* regulate *bla*_OXA-58-like_ gene expression. Meanwhile, the latter four all provide hybrid promoters, as described in the recent studies [5, 6, 8, 29]. IS*1008*-ΔIS*Aba3* was the most common IS upstream of the *bla*_OXA-58-like_ gene in *A*. *baumannii* clinical isolates in this study, followed by IS*Aba3* and IS*Aba1*. Chen et al. reported that a single plasmid-borne IS*1008*-ΔIS*Aba3*-*bla*_OXA-58_ is enough to confer a high level of resistance to carbapenem for *A*. *baumannii*. The insertion of IS*1008* provided a hybrid promoter and increased the transcription level of the *bla*_OXA-58_ gene [8]. However, the present study found that IS*1008*-ΔIS*Aba3*-*bla*_OXA-58-like_-harboring *A*. *baumannii* isolates showed variable susceptibility to carbapenems (MICs 1 μg/ml to 32 μg/ml). Meanwhile, the similar carbapenem MIC distributions were also detected in IS*Aba3*-*bla*_OXA-58-like_-harboring *A*. *baumannii* isolates (MICs 0.5 μg/ml to 32 μg/ml). The reasons for the variation in the resistance levels remain unknown. Several previous studies demonstrated that the overexpression of the AdeABC efflux pump and expression of OXA-23 or OXA-58 lead to higher levels of carbapenem resistance [26, 30–32]. In addition, Bertini et al. [33] described that the multiple copies of *bla*_OXA-58_ increase the level of resistance to carbapenems. However, the study of D'Arezzo showed the opposite conclusion; they reported that the resistance to meropenem or imipenem is not associated with *bla*_OXA-58-like_ gene copy number per plasmid or to loss of integrity of the CarO porin [26]. Taken together, we speculate that the variable ISs upstream of the *bla*_OXA-58-like_ gene, high copy number of *bla*_OXA-58-like_, overexpression of efflux system, and other cofactors may confer a high level of resistance to carbapenem in *A*. *baumannii*. In addition, neither IS*Aba1* nor IS*Aba4* was detected upstream of the *bla*_OXA-23-like_ gene in one isolate in the current study, though several attempts were conducted. This result may be due to another unknown resistance mechanism, which confers resistance to carbapenems in this isolate.

*A*. *baumannii* clonal complex 92, corresponding to the global clone 2, has been found worldwide [34], which comprises more than 100 STs, including ST75, ST92, ST92/ST208, and ST381. To the best of our knowledge, ST75, ST92, and ST92/ST208 were the most common STs in China, and ST381 was first identified as sporadic clone in two hospitals in Sichuan, Southwest China in 2011. All of these STs harbored *bla*_OXA-23_ gene [11, 12]. Notably, the present study demonstrated the prevalence of ST92/ST208 with *bla*_OXA-58-like_ in Shenzhen People’s Hospital prior to 2008. Surprisingly, ST381 with *bla*_OXA-23-like_ first emerged in this hospital on December 30, 2008; thereafter, it rapidly spread and replaced the ST92/ST208 and ST229 with *bla*_OXA-23-like_ in 2009. ST229 occurred for the first time in this hospital in 2006 and became one of the main clones in 2007 and 2008, which is genetically completely unrelated to ST92/ST208 and ST381. The reasons for the prevalence of clone J (ST381) are still unknown. Clone J was first isolated from the sputum of a 74-year-old male diabetes inpatient, who had been artificially ventilated for seven days in intensive care unit (ICU) for severe community-acquired pneumonia. The reinfection of *A*. *baumannii* was confirmed by infectious-disease physicians with subsequent several positive sputum cultures, clinical symptoms and signs, and effective responses to antibiotic therapy against *A*. *baumannii* with cefoperazone-sulbactam. This patient impossibly introduced the ST381 strain with *bla*_OXA-23-like_ in the hospital, because both of his two sputum cultures obtained on the first and fifth days of his hospitalization in the ICU showed negative results. This strain possibly survived in the ICU environment prior to this infection. By investigating the usage of carbapenems in the inpatients of Shenzhen People’s Hospital from 2004 to 2009, 1.8 and 2.2-fold increase of defined daily doses (DDDs) of imipenem (1521.25 to 2777.5) and meropenem (1464.75 to 3232.5) were observed in 2009, respectively. In particular, the DDDs of meropenem had been maintained higher than those of imipenem since 2006 (S1 Fig). Neither a change in the hospital policy nor the introduction of a new antibiotic was observed during this period. We hypothesized that the increasing selective pressure in this hospital environment screened the clone J with *bla*_OXA-23-like_, which subsequently caused the huge outbreak in 2009. Minandri et al. [10] investigated the transition of *bla*_OXA-58_ to *bla*_OXA-23_ gene carriage from 2005 to 2009 among *A*. *baumannii* isolates responsible for ICU outbreaks in the main hospitals of central Italy. They found that all isolates from the transition period demonstrate extensive genetic similarity, all belonging to ST2 determined by the scheme of Daincourt et al [35]. Interestingly, the present study also indicates the occurrence of clone replacement between genetically similar ST381 and ST92/ST208. We speculate that the higher carbapenemase activity of OXA-23-like compared with OXA-58-like, may provide *bla*_OXA-23-like_-carrying ST 381with a selective advantage over *bla*_OXA-58-like_-carrying ST92/ST208 by increasing the resistance to both imipenem and meropenem. However, the dominant role of ST 381 remains unknown among the *A*. *baumannii* population in the short period other than ST229, although the latter occurred earlier. Further study is needed to elucidate this question.

## Conclusion

We first reported the distinct *bla*_OXA-23-like_-carrying *A*. *baumannii* ST381 with high level of resistance to carbapenems, which rapidly spread and replaced the previously prevalent *bla*_OXA-58-like_-carrying ST92/ST208 with variable susceptibility to carbapenems, resulting in the increased resistance to carbapenems in *A*. *baumannii* in a Chinese hospital in 2009.

## Supporting Information

S1 Fig DDDs of imipenem and meropenem from 2004 to 2009. (TIF)
