# Supplementary figures and images for: Transition of bla OXA-58-like to bla OXA-23-like in Acinetobacter baumannii Clinical Isolates in Southern China: An 8-Year Study
Source: PLoS One. 2015 Sep 4;10(9):e0137174. doi: 10.1371/journal.pone.0137174 (PMC4560421; doi:10.1371/journal.pone.0137174)

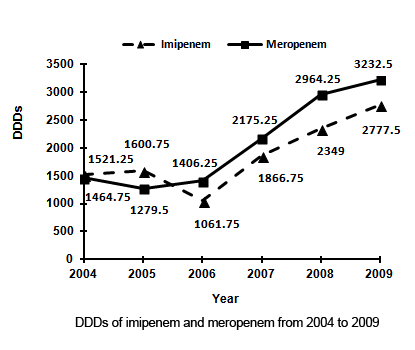

Supplement: S1 Fig — (TIF) [file pone.0137174.s001.tif]
